# COVID-19-Related Trends and Characteristics of Type 2 Diabetes Mellitus and Metabolic Syndrome

**DOI:** 10.7759/cureus.21483

**Published:** 2022-01-21

**Authors:** Brittany N Franco, Shinichi Asano

**Affiliations:** 1 Department of Biomedical Sciences, West Virginia School of Osteopathic Medicine, Lewisburg, USA

**Keywords:** blood glucose, hemoglobin a1c, covid pandemic, metabolic syndrome, type 2 diabetes mellitus

## Abstract

Background

Although there have been several studies associating obesity with severe coronavirus disease 2019 (COVID-19) outcomes, the potential impact of the pandemic on type 2 diabetes mellitus (T2DM) and metabolic syndrome (MetS) incidence is less clear. Furthermore, reports on the characteristics of these patients during the pandemic have been scarce.

Objectives

The purpose of this retrospective study was 1) to explore the impact of the COVID-19 pandemic on T2DM and MetS incidence, and 2) to describe sex-based differences in the characteristics of T2DM and MetS patients during the COVID-19 pandemic.

Methods

Using electronic health records (EHRs) obtained from the USA-based TriNetX research database (TriNetX, Cambridge, MA), the incidence and the total number of patients with “T2DM (ICD-10-CM: E11)” and “MetS (ICD-10-CM: E88.81)” prior to and during the COVID-19 pandemic were determined. Aggregate lab data from EHRs were extracted and statistical analyses on the lab values and patient demographics including sex, race/ethnicity, and comorbidities were performed.

Results

After analyzing T2DM and MetS patient data from 2018, 2019, and 2020, we observed a considerable decrease in both T2DM and MetS incidence, with data in April 2020 exhibiting the largest decrease when compared to other months. Furthermore, monthly male T2DM and MetS patients’ lab data revealed worsening parameters in April 2020, such as hemoglobin A1c (HbA1C) and blood glucose, when compared to females. Demographic data during 2020 revealed that male T2DM and MetS patients had a significantly higher prevalence of comorbidities including hypertension, ischemic heart disease, and heart failure, but female T2DM and MetS patients had significantly higher asthma comorbidity.

Conclusions

During 2020, there was a marked decrease in T2DM and MetS diagnosis. Due to a lack of screening, these data may suggest a subsequent increase in T2DM and MetS-related heart disease in the future and may magnify the existing sex-related differences identified in these patients.

## Introduction

The Centers for Disease Control and Prevention (CDC) states that over 1.5 million Americans per year are diagnosed with type 2 diabetes mellitus (T2DM), which is characterized by increased insulin resistance and blood glucose levels, and decreased insulin secretion [1]. Similarly, metabolic syndrome (MetS) is characterized by a group of related disorders including obesity, dyslipidemia, hyperglycemia, and hypertension [2], and it is estimated that the prevalence of MetS was 34.7% among United States adults based on the National Health and Nutrition Examination Survey (NHANES) data from 2011 through 2016 [3]. As a result of the poor diet choices and sedentary lifestyle seen in our society, the number of patients with T2DM and MetS is expected to increase further. Due to the increased risk that both T2DM and MetS patients have for developing cardiovascular disease (CVD), frequent health screening and disease management are critical in the prevention of these major obesity-related life-threatening events such as heart attack and stroke. Furthermore, recent data suggest that sex is an important biological variable in CVD, and that sex and obesity interact to produce either protective or detrimental effects on cardiovascular function [4,5]. 

Since the emergence of the coronavirus disease 2019 (COVID-19) pandemic, the priority of limiting the spread of the virus has superseded the importance of screening for chronic diseases. In fact, a significant decrease in cancer screenings and new diagnoses was reported during the spring of 2020, which may raise concerns about potential increases in advanced stages of cancer diagnosis in the near future [6]. Also, a recent report indicated that patients with chronic diseases are more uncomfortable going to the hospital due to the anxiety of acquiring a COVID-19 infection [7]. Due to the psychological fear of a COVID-19 infection and unfamiliar COVID-19-related protocols in healthcare settings, patients may have been reluctant to make routine visits to their primary care physicians. Throughout 2020, the COVID-19 pandemic had forced the healthcare industry to prioritize the treatment of COVID-19 patients. This had a significant impact not only on clinical practices but also on the perception as to when and why a person should seek medical care.

In an attempt to gain pathophysiological insights into the effects of obesity on COVID-19 infection, there have been numerous studies reporting that obesity is associated with an increase in the incidence and severity of COVID-19 [8-10]. However, the impact of the pandemic on T2DM and MetS diagnosis and screening is less clear. Also, risk stratification by sex and T2DM/MetS characteristics play a key role in the reduction of premature CVD mortality [11], but there is limited information on sex disparities present in COVID-19 pandemic-related T2DM and MetS screenings such as blood glucose and hemoglobin A1C (HbA1C). In light of this, the objective of the current study is 1) to investigate the impact of the COVID-19 pandemic on T2DM and MetS incidence, and 2) to describe sex-based differences in the characteristics of T2DM and MetS during the COVID-19 pandemic.

A part of this data was previously presented at the 2021 American Physiological Society “New Trends in Sex and Gender Medicine” Conference on October 19-22, 2021.

## Materials and methods

Data source

Aggregate data from electronic health records (EHRs) were obtained from a subset of healthcare organizations (HCOs) that are associated with the TriNetX research network (TriNetX, Cambridge, MA) as described previously [12-14]. TriNetX is a global federated health research network that provides access to real-time EHR data, including demographics, diagnoses, laboratory results, and vital signs. The HCOs associated with the TriNetX network comprise private practices, community hospitals, and academic medical centers [15]. This particular study included HCOs across the United States. TriNetX has a waiver from the Western Institutional Review Board since only aggregated counts and statistical summaries of de-identified patient information are used and no protected health information is received. This study was also reviewed by the West Virginia School of Osteopathic Medicine (WVSOM) Institutional Review Board and received a waiver.

Study protocols

We categorized our search primarily into two groups of patients: 1) those presenting to an HCO with the diagnosis of T2DM and 2) those who presented to an HCO with the diagnosis of MetS. The queries for this retrospective study were made using the diagnostic terms: “type 2 diabetes mellitus (ICD-10 CM: E11)” and “metabolic syndrome (ICD-10-CM: E88.81)”. These queries were made during a specified time frame to compare results prior to and during the COVID-19 pandemic (e.g., April 1, 2019, to April 30, 2019, vs. April 1, 2020, to April 30, 2020). This process was repeated for each month of 2018, 2019, and 2020. The incidence was described as the number of new diagnoses observed within each time frame. The percentage of changes was calculated by normalizing the data in 2018 from the same month. For the 2020 patient demographic data and monthly lab data, the same procedure was used to select the first diagnosis of T2DM or MetS data using a specific time frame as detailed above.

Data analysis

After patient data were extracted, male and female data were separated, and a comparison was made using TriNetX analytical features. For patients’ demographic data including sex, race, and comorbidities, contingency tables were created and the frequencies in the groups were analyzed by the chi-square test using the TriNetX analytical function. The comorbidities listed in Tables 1, 2 were selected because these conditions are often associated with T2DM and MetS patients, and similar analyses had also been utilized in a previous study using the TriNetX database [16]. Extracted lab data were analyzed by unpaired t-tests using GraphPad Prism statistical software (GraphPad Software Inc., San Diego, CA). These data are presented as means and standard deviation (mean ± SD).

## Results

Number of patients with T2DM and incidence

**Figure 1:**
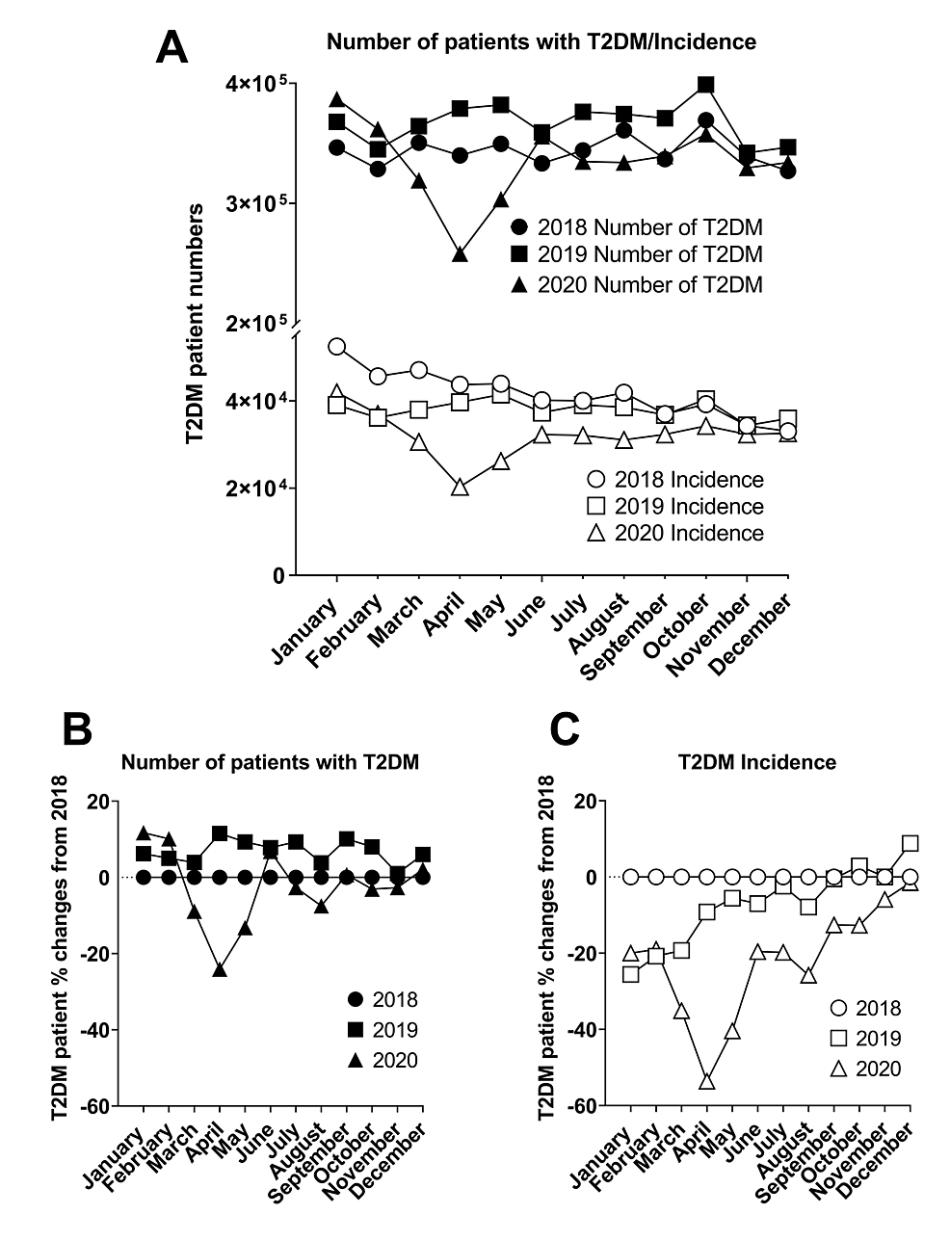
Type 2 diabetes mellitus (T2DM) patients seen in a healthcare setting during the COVID-19 pandemic Panel A: Total number of T2DM patient visits, and first-time T2DM-diagnosis patients (incidence) in 2018, 2019, and 2020. Panel B: Normalized number of patients with T2DM during the pandemic using 2018 counts as a control. Panel C: Normalized T2DM patient incidence during the pandemic using 2018 counts as a control COVID-19: coronavirus disease 2019

From January to December 2018, the average monthly number of patients with T2DM was 343,768 ± 12,568. Similarly, the average monthly number of patients with T2DM was 367,216 ± 16,866 in 2019. However, in 2020, the average monthly number of patients with T2DM was 334,538 ± 32,441. Specifically, the number of patients with T2DM was up to 24.1% lower than the 2018 data. Furthermore, there was a 31.9% decline in the number of patients with T2DM noted for the month of April 2020 when compared to April 2019 (379,014 vs. 258,086 in 2019 and 2020, respectively). These data demonstrate that the number of patients with T2DM differed prior to and during the COVID-19 pandemic (Figures 1A, 1B).

From January to December 2018, the average monthly T2DM patient incidence was 41,540 ± 5,497. Similarly, the average monthly T2DM patient incidence in 2019 was 38,059 ± 2,045. However, in 2020, the average monthly T2DM patient incidence was 31,910 ± 5,253. Specifically, T2DM patient incidence was up to 53.6% lower than the 2018 data. These data exhibit the difference in incidence prior to and during the COVID-19 pandemic (Figures 1A, 1C). Taken together, these data suggest that both the total number of patients with T2DM and the first incidence of T2DM patients dramatically decreased during the COVID-19 pandemic. Interestingly, the first incidence of T2DM patients’ data appeared to be more affected than existing T2DM patients’ data (Figures 1B, 1C).

Number of patients with MetS and incidence

**Figure 2:**
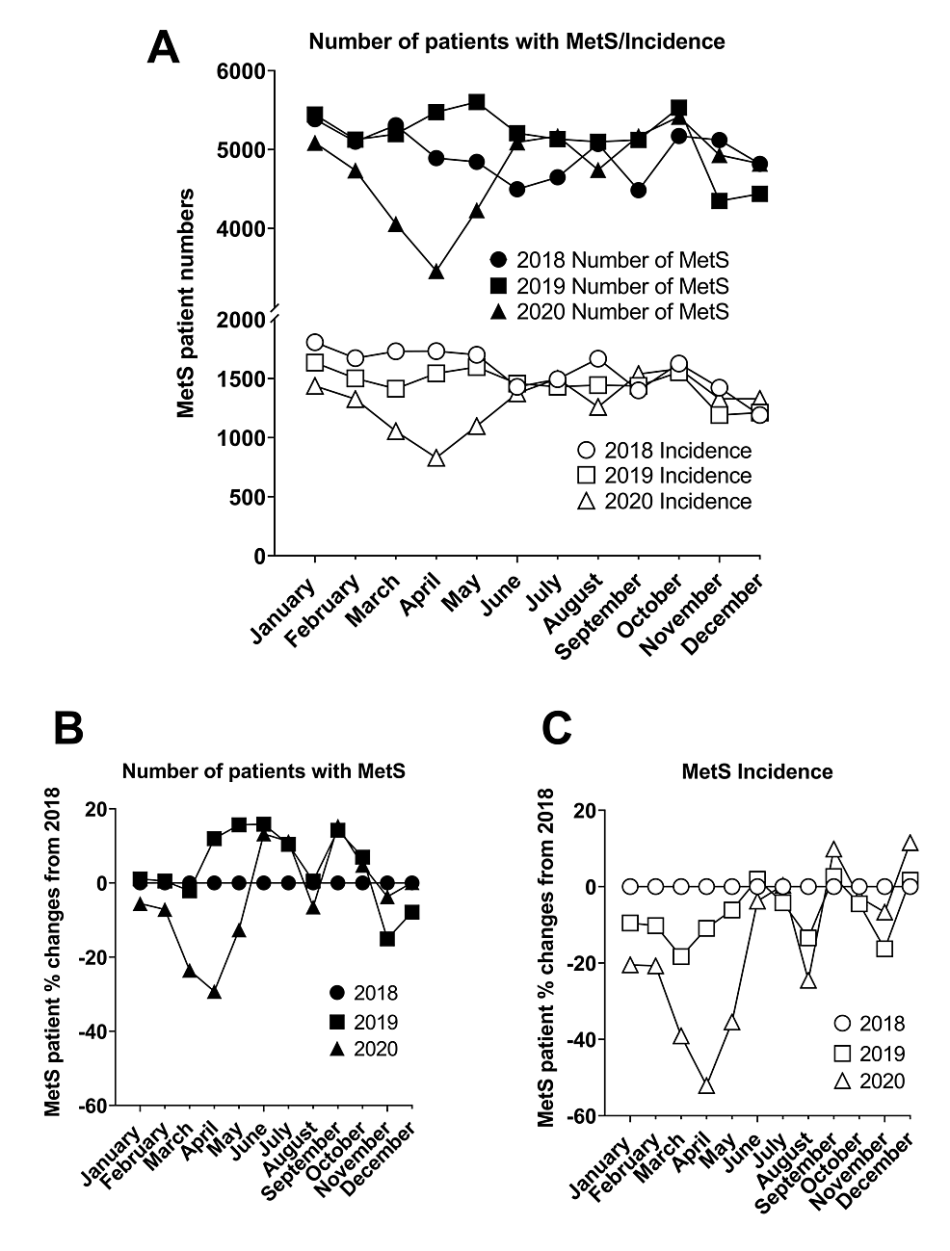
Metabolic syndrome (MetS) patients seen in a healthcare setting during the COVID-19 pandemic. Panel A: Total number of MetS patient visits, and first-time MetS-diagnosis patients (incidence) in 2018, 2019, and 2020. Panel B: Normalized number of MetS patients during the pandemic using 2018 counts as a control. Panel C: Normalized MetS patient incidence during the pandemic using 2018 counts as a control COVID-19: coronavirus disease 2019

From January to December 2018, the average monthly number of patients with MetS was 4,945 ± 298. Similarly, the average monthly number of patients with MetS was 5,144 ± 393 in 2019. However, in 2020, the average monthly number of patients with MetS was 4,743 ± 562. Specifically, the number of patients with MetS was up to 29.2% lower than the 2018 data. Furthermore, there was a considerable difference in the number of patients with MetS noted for the month of April: 4,891, 5,475, and 3,461 in 2018, 2019, and 2020, respectively. These data demonstrate that the number of patients with MetS differed prior to and during the COVID-19 pandemic (Figures 2A, 2B).

From January to December 2018, the average monthly MetS patient incidence was 1,573 ± 183. Similarly, the average monthly MetS patient incidence in 2019 was 1,451 ± 136. However, in 2020, the average monthly MetS patient incidence was 1,304 ± 219. Specifically, MetS patient incidence was up to 52.1% lower than the 2018 data. These data exhibit the difference in incidence prior to and during the COVID-19 pandemic (Figures 2A, 2C). Similar to the T2DM data, these data suggest that both the number of patients with MetS and the first incidence of MetS patients dramatically decreased during the COVID-19 pandemic. Interestingly, the first incidence of MetS patients’ data also appeared to be more affected than the number of patients with MetS data (Figure 2B, 2C).

2020 T2DM patient demographics

Demographic and clinical characteristics of patients are described in Table 1. Demographic data during 2020 revealed a total of 377,809 T2DM patients with a statistically higher number of male T2DM patients who self-identified as white compared to female T2DM patients (male vs. female: 63% vs. 59%, p<0.001). Contrastingly, there was a significantly lower number of male T2DM patients who self-identified as black compared to T2DM females (male vs. female: 16% vs. 21%, p<0.001). Additionally, male T2DM patients had a significantly higher prevalence of comorbidities including hypertension (male vs. female: 66% vs. 64%, p<0.001), ischemic heart disease (male vs. female: 27% vs. 17%, p<0.001), and heart failure (male vs. female: 14% vs. 11%, p<0.001) as described in Table 1. On the other hand, female T2DM patients had significantly higher asthma comorbidity (male vs. female: 6% vs. 12%, p<0.001).

**Table 1:** Type 2 diabetes mellitus (T2DM) patient demographics from January to December 2020 The frequencies in the groups were analyzed by the chi-squared test; the entries I10, N18, etc. represent the ICD-10 diagnostic codes COPD: chronic obstructive pulmonary disease; ICD-10: International Classification of Diseases, Tenth Revision; SD: standard deviation

T2DM incidence, January-December 2020							
	Diagnosis		Male		Female	P-value	
Number of patients (n)			193,963		183,846		
Age in years, mean ± SD			61.1 ± 14.8		60.4 ± 16.2	<0.0001	
Race							
White (%)			63		59	<0.0001	
Black or African American (%)			16		21	<0.0001	
Unknown (%)			17		16	<0.0001	
Asian (%)			3		3	0.9683	
American Indian or Alaskan Native (%)			1		1	0.104	
Comorbidities							
Hypertension (%)	I10		66		64	<0.0001	
Ischemic heart disease (%)	I20-I25		27		17	<0.0001	
Chronic kidney disease (%)	N18		17		14	<0.0001	
Acute kidney failure (%)	N17		15		10	<0.0001	
Heart failure (%)	I50		14		11	<0.0001	
Atrial fibrillation and flutter (%)	I48		12		8	<0.0001	
COPD (%)	J44		9		9	0.8492	
Asthma (%)	J45		6		12	<0.0001	
Overweight and obesity (%)	E66		26		33	<0.0001	

2020 MetS patient demographics

Similarly, demographic data during 2020 revealed a total of 15,841 MetS patients, with a statistically higher white population of males compared to female patients (male vs. female: 70% vs. 64%, p<0.001). Contrastingly, there was a significantly lower African American male population compared to African American females (male vs. female: 13% vs. 20%, p<0.001). Male MetS patients also had a significantly higher prevalence of comorbidities including hypertension (male vs. female: 66% vs. 51%, p<0.001), ischemic heart disease (male vs. female: 26% vs. 12%, p<0.001), and heart failure (male vs. female: 13% vs. 7%, p<0.001) as described in Table 2. On the other hand, female MetS patients had significantly higher asthma comorbidity (male vs. female: 13% vs. 21%, p<0.001).

**Table 2:** Metabolic syndrome (MetS) patient demographics from January to December 2020 The frequencies in the groups were analyzed by the chi-squared test; the entries I10, N18, etc. represent the ICD-10 diagnostic codes COPD: chronic obstructive pulmonary disease; ICD-10: International Classification of Diseases, Tenth Revision; SD: standard deviation

MetS incidence, January-December 2020								
	Diagnosis	Male		Female			P-value	
Number of patients (n)		5,819		10,022				
Age in years, mean ± SD		51.4 ± 18.8		46.4 ± 18.2			<0.0001	
Race								
White (%)		70		64			<0.0001	
Black or African American (%)		13		20			<0.0001	
Unknown (%)		14		13			0.0616	
Asian (%)		2		2			0.3245	
American Indian or Alaskan Native (%)		1		1			0.1654	
Comorbidities								
Hypertension (%)	I10	66		51			<0.0001	
Ischemic heart disease (%)	I20-I25	26		12			<0.0001	
Chronic kidney disease (%)	N18	17		9			<0.0001	
Acute kidney failure (%)	N17	16		7			<0.0001	
Heart failure (%)	I50	13		7			<0.0001	
Atrial fibrillation and flutter (%)	I48	11		5			<0.0001	
COPD (%)	J44	9		7			<0.0001	
Asthma (%)	J45	13		21			<0.0001	
Overweight and obesity (%)	E66	60		65			<0.0001	

2020 T2DM and MetS lab values

**Figure 3:**
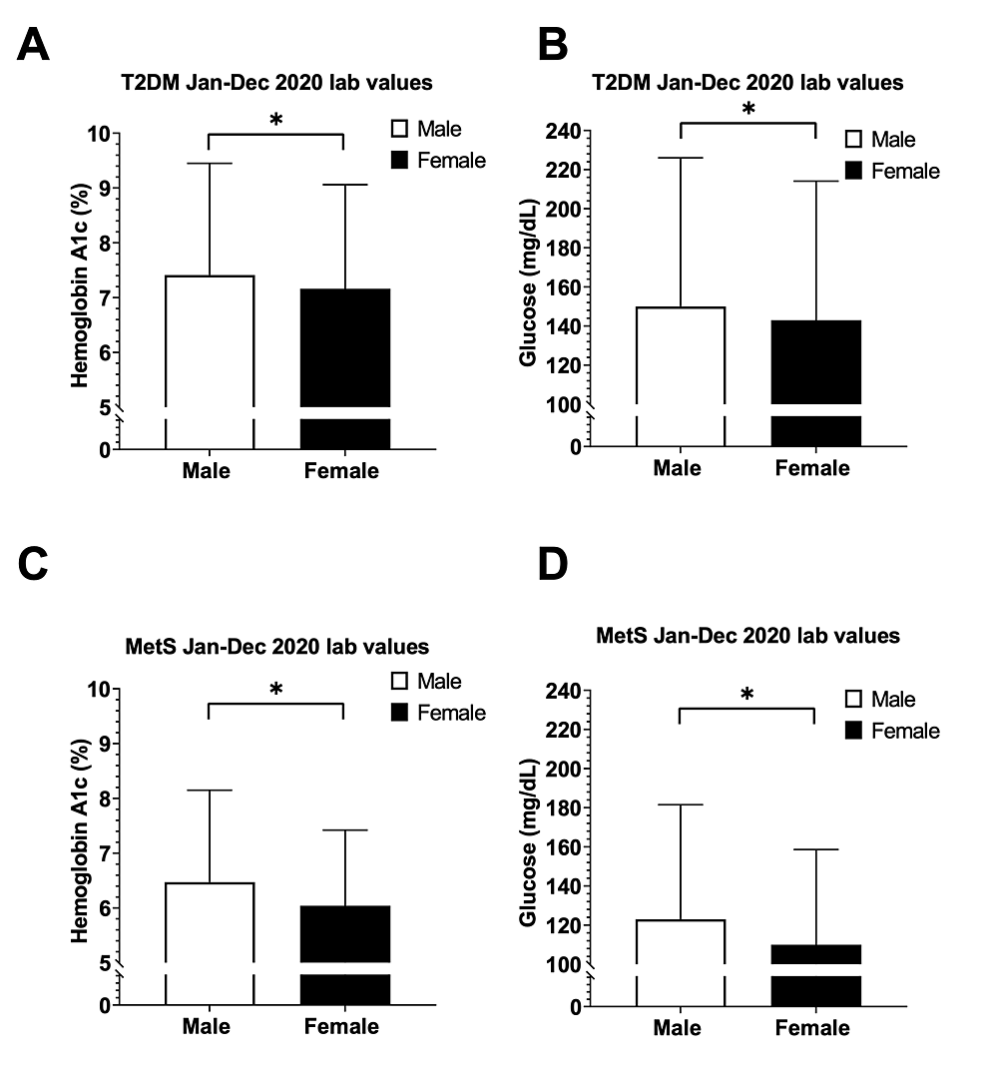
HbA1C and blood glucose values of male vs. female type 2 diabetes mellitus (T2DM) and metabolic syndrome (MetS) patients from January to December 2020 Panel A: Elevated HbA1C in male T2DM patients (n=106,655) compared to female T2DM patients (n=102,907) from January to December 2020. Panel B: Elevated blood glucose in male T2DM patients (n=143,914) compared to female T2DM patients (n=138,151) from January to December 2020. Panel C: Elevated HbA1C in male MetS patients (n=4,111) compared to female MetS patients (n=7,189) from January to December 2020. Panel D: Elevated blood glucose in male MetS patients (n=5,003) compared to female MetS patients (n=8,500) from January to December 2020 Error bars indicate standard deviation. *Indicates significant differences between male vs. female by an unpaired t-test, p<0.01 HbA1C: hemoglobin A1c

**Figure 4:**
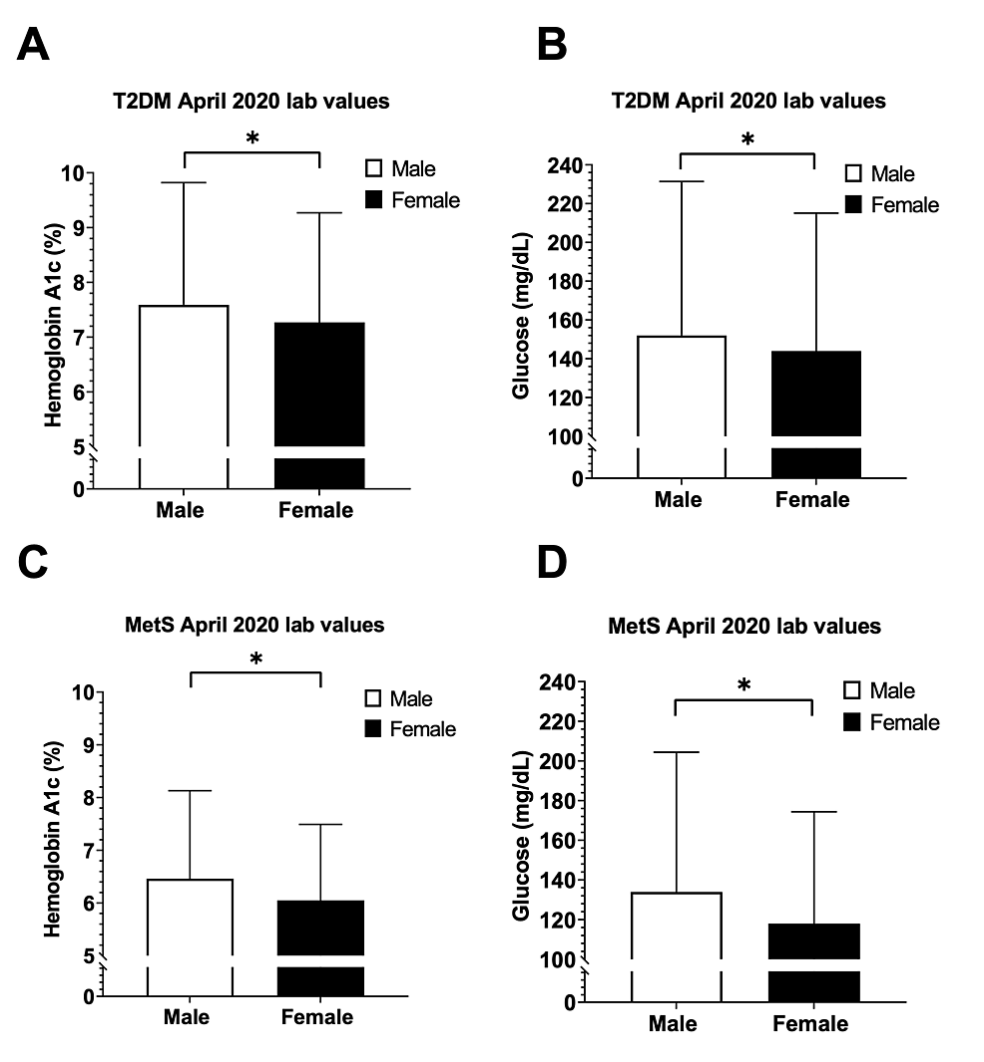
HbA1C and blood glucose values of male vs. female type 2 diabetes mellitus (T2DM) and metabolic syndrome (MetS) patients in April 2020 Panel A: Elevated HbA1C in male T2DM patients (n=6,049) compared to female T2DM patients (n=5,339) in April 2020. Panel B: Elevated blood glucose in male T2DM patients (n=8,219) compared to female T2DM patients (n=7,313) in April 2020. Panel C: Elevated HbA1C in male MetS patients (n=230) compared to female MetS patients (n=374) in April 2020. Panel D: Elevated blood glucose in male MetS patients (n=275) compared to female MetS patients (n=445) in April 2020 Error bars indicate standard deviation. *Indicates significant differences between males vs. females by an unpaired t-test, p<0.01 HbA1C: hemoglobin A1c

To explore the severity of T2DM and MetS between males and females, we compared HbA1C and blood glucose lab values from January to December 2020 (Figure 3). Given that April 2020 showed the most significant decline in T2DM and MetS incidence, we examined the corresponding HbA1C and blood glucose lab values for that patient cohort. Our analysis displayed a marked increase in severity for the month of April 2020 in both T2DM and MetS patients (Figure 4). The mean HbA1C and blood glucose values were significantly higher in the male T2DM patients compared with the female T2DM patients (HbA1C: male: 7.59 ± 2.24 vs. female: 7.27 ± 2, p<0.001; glucose: male: 152 ± 79.4 vs. female: 144 ± 71.7, p<0.001). The same trend was observed in MetS patients.

## Discussion

The purpose of this study was to explore if the COVID-19 pandemic affects the incidence of T2DM and MetS and to describe the characteristics of these patients during the ongoing pandemic. Although the majority of previous studies have suggested that the characteristics of T2DM and MetS such as obesity, hypertension, dyslipidemia, and hyperglycemia are associated with an increase in the incidence and severity of COVID-19 [8-10], there is little information in the literature to indicate that the diagnosis of T2DM and MetS was affected by the COVID-19 pandemic. In this study, we described the disparate phenomena of both the incidence and the total number of T2DM and MetS patients in 2020 compared to the two previous years (2018 and 2019). Specifically, in our analysis, there was a noticeable decrease in new T2DM diagnosis, as well as the total number of T2DM patients, with April 2020 showing a 54% decrease in incidence and a 24% decrease in the total number of T2DM patients compared to April 2018 (Figure 1). Similarly, there was a marked decrease in new MetS diagnosis, as well as the total number of patients with MetS in April 2020 (Figure 2). These trends are consistent with a recent cancer diagnosis study that described significant declines in both existing and new incidence of different types of cancer from January 2020 to April 2020 when compared to January 2019 to April 2019 [6]. Thus, our data provide additional evidence that COVID-19 not only caused a health crisis by direct infection, but also by the indirect effects of the pandemic. Interestingly, we did not observe any noticeable rebound or increase in T2DM and MetS incidence throughout the rest of 2020. Currently, it is unclear if these declines in newly diagnosed T2DM and MetS patients we observed were due to physician burn-out, lack of healthcare providers, fear of visiting a hospital, or some other factors, and hence warrant further investigation. Also, whether these pandemic-related declines in screening and diagnoses of T2DM and MetS patients will affect the incidences of T2DM associated life-threatening cardiovascular events in the future is difficult to predict. Further studies are needed to closely monitor these populations who are at considerable risk of developing CVD in the future.

It is clear that due to the COVID-19 pandemic, the number of T2DM and MetS diagnoses in 2020 differed from the previous years; moreover, another interesting finding of our analyses was the differing severity of T2DM and MetS patients between males and females from January to December 2020 (Figure 3). Specifically, April 2020 data showed that males exhibited worsening HbA1C and blood glucose values among both T2DM and MetS patients (Figure 4). Of particular interest is our finding that MetS patient demographics in 2020 appeared to be different according to sex. Specifically, cardiovascular-related comorbidities such as hypertension, ischemic heart disease, and heart failure were significantly more frequent in male patients than female patients. Our analysis also revealed the racial disparity in both T2DM and MetS patients with the white male population being significantly higher when compared to female patients, whereas the opposite trend was seen with the African American population. Although sex is recognized as a critical biological variable for obesity-related CVD [4,5], these sex and racial disparities present in 2020 T2DM and MetS patients may magnify the existing sex differences in heart attack and stroke risk in the future. This also emphasizes the importance of closely monitoring future T2DM and MetS patients and minimizing the effect that the COVID-19 pandemic has on the screening and treatment of these patients.

Limitations

Although TriNetX obtains aggregate data from millions of patients, there are patients who have been seen in other HCOs that are not within the TriNetX research network. Also, our data subscription would not allow us to perform a fully adjusted descriptive statistical analysis. Thus, these data and our analysis should not be simply generalized to the wider population; however, the data samplings cover over 40 HCOs rather than a single HCO, and the data extraction methods such as timely obtained EHRs are consistent.

## Conclusions

The COVID-19 pandemic has had a substantial impact on individuals and healthcare systems worldwide. We described a significant decrease in T2DM and MetS incidence during the pandemic, which may suggest the possibility of a subsequent increase in the diagnosis of T2DM and MetS-related heart disease. This clear decline in T2DM and MetS incidence is most likely a multifactorial process, but the repercussions of it may be observed in the future.
